# Editorial: Single-Molecule Image Analysis

**DOI:** 10.3389/fbinf.2022.897938

**Published:** 2022-04-06

**Authors:** Thomas Pengo, Siân Culley, Christian Franke

**Affiliations:** ^1^ Informatics Institute, University of Minnesota, Minneapolis, MN, United States; ^2^ Randall Centre for Cell and Molecular Biophysics, School of Basic and Medical Biosciences, Faculty of Life Sciences and Medicine, King’s College London, London, United Kingdom; ^3^ Institute of Applied Optics and Biophysics, Faculty of Physics and Astronomy, Friedrich Schiller University Jena, Jena, Germany; ^4^ Abbe Center of Photonics, Friedrich Schiller University Jena, Jena, Germany; ^5^ Jena Center for Soft Matter, Friedrich Schiller University Jena, Jena, Germany

**Keywords:** single-molecule localisation microscopy, SMLM, super-resolution microscopy, image analysis, clustering, tracking, software

Seeing is believing. This little sentence illustrates the prime reason why microscopy is such a widespread and powerful tool for the study of biological systems. To observe something with one’s own eyes, even with the help of a microscope, is a potent experience. The super-resolved approach single-molecule localization microscopy (SMLM), however, goes beyond the qualitative aspects of biological imaging at an unprecedented level of detail: it offers the unique ability to capture spatio-temporal coordinates for detected fluorescence emission events from individual molecules. Today, SMLM is applied increasingly as a quantitative tool for, e.g., molecular clustering, tracking and stoichiometry, benefitting from the multitude of extractable parameters within the set of localized coordinates.

In this research topic we have gathered contributions from scientists working on a broad range of aspects in SMLM, all unified in their focus on extracting information from multi-dimensional localization data. In total, 12 articles have been included, with a mixture of 10 original research articles (2 technology and code, 5 original research, 3 methods), 1 review and 1 opinion article.

Since we started working on the research topic, we all have witnessed a very difficult year. The pandemic is claiming and changing the lives of countless people around the world, hitting the already disadvantaged worst and exacerbating the painful reality of disparity between those who have access to wealth and those who don’t. Meanwhile, other diseases still persist, and sadly last year saw the loss of one of the SMLM community’s most respected leaders, Kat Gaus, to cancer. In his special contribution to the research topic Cebecauer offers a heartfelt tribute to her life and a glimpse into the rich legacy she left behind.

Since its first description in the literature a decade and a half ago, SMLM has flourished into a well-established method, albeit one that is difficult to master due to its reliance on specialized fluorophores, stringent illumination requirements, and intensive data processing.

Here, Martens et al. have demystified the computational aspect of SMLM by producing a wonderful summary of the processing required to bring a dataset from raw data to analysis and visualization. In their work, the authors provide not only a general introduction to the methods, divided in eight modules, but also ready-to-use educational code and tutorials in both MATLAB and Python.

One of the key computational challenges in SMLM is extracting biologically meaningful information from molecular coordinates. A number of articles in this research topic are thus dedicated to tackling the analysis of multi-dimensional spatial localization distributions.

In an ideal SMLM experiment, a single fluorophore blinking event would be detected within one camera frame. However, in reality, one individual emission event can span multiple camera frames; without appropriate analytical treatment this can result in over-counting the number of emitting molecules. Here, Schodt and Lidke present a molecularly-informed grouping algorithm, formulated as a linear assignment problem, that accounts for photophysical kinetics, e.g., multiple blinking steps and bleaching, and local emitter density variations. This helps assign detected emission events to the correct fluorophore.

Structural resolution is one of the key parameters in SMLM experiments and is a multivariate problem, since not only the localization precision of single fluorophores, but also the final localization density is significant. Especially in experimental settings, where resolution is mainly limited by the resulting localization density, e.g., in expansion microscopy, backfilling the image by deep-learning approaches has become a promising approach. In their manuscript, Berberich et al. describe a clever approach of utilizing Fourier Ring Correlation as a trainable quality metric that can be based on sparse SMLM data sets. This new loss function can complement a multiscale structural similarity index, leading to an overall improved reconstruction and can be easily integrated in existing deep-learning and analysis workflows.

SMLM data is not limited to just two dimensions, and several articles here describe analytical methods relating to extension of SMLM in the axial, temporal, and spectral dimensions.

In an interesting overlap with computer vision, Blundell et al. show how it is possible to use Deep Learning to perform what is otherwise a hard problem: finding 3D structure from individual 2D projections. HOLLy, their software, uses a Convolutional Neural Network, coupled to a differentiable renderer, to explore the space of 3D structures and find candidates that match the input data.

The single molecule nature of SMLM dovetails into the field of single particle tracking (SPT), whereby the temporal dynamics of labelled protein species can be measured. In order to increase the throughput of SMLM for tracking approaches, Butler et al. designed a microscope capable of spectrally resolving 3D localizations in dynamic samples, allowing for identification of different fluorophores without imaging in multiple channels. Alongside this hardware development, they showcase an analytical framework for disentangling the multi-dimensional data acquired and leveraging spectral information to inform multi-emitter fitting analysis.

SPT data from living samples presents a complex analysis problem for dissecting contributions from biological environments with an unknown number of diffusive states. Chen et al. combine Deep Learning with non-parametric Bayesian inference to tackle complicated live-SPT data for which: “the number of diffusive states is unknown, mixtures of different diffusive populations may exist within single trajectories, symmetry cannot be assumed between the *x* and *y* directions, and anomalous diffusion is possible.”

Many signalling pathways in cell biology are mediated by protein clustering at the cell membrane. As SMLM provides the coordinates of individual molecules, it is an ideal technique for quantifying the spatial distributions of such clusters. This quantification is enabled by Kutz et al., who provide access to advanced Bayesian cluster analysis algorithms through a Graphical User Interface, BaClAva. This software not only supports the analysis of SMLM data, but also simulation, through which the user can explore the effect of algorithm and parameter choice on clustering performance.

Cluster analysis is a challenging problem not just within the context of a single image, but also when aggregating results across different datasets. Feher et al. have developed a novel algorithm called KNA (K-neighborhood analysis), based on principal component analysis of vectors between nearest-neighbor molecule coordinates. The authors demonstrate the application of this approach in enhancing the performance of existing clustering algorithms, visualising SMLM data in terms of molecule neighborhood, and joint analysis across distinct datasets. Beyond these technical advances, this manuscript is particularly remarkable as it was finalized in a spirited manner by Kat Gaus‘ group after her death in March 2021.

In addition to uncovering whether molecules of the same species form clusters, extending SMLM into multiple colors can provide information on interactions between different molecular species. Mancebo et al. describe a novel method for efficiently isolating clusters which co-localize with a second channel, based on using k-dimensional trees. This method, implemented in MATLAB, offers a dramatic reduction in memory and computational power requirements over other methods.

One of the topics the scientific community is particularly concerned about at the moment is reproducibility. In this research topic, we had contributions that tackled the issue of how far we can trust the results, either by quantifying the uncertainty or, complementarily, how to integrate quality metrics to improve the final resolution.

Thiele et al. present a theoretical discourse of the estimation of the fluorescence lifetime in FLIM and SML-FLIM. By using a maximum likelihood estimator, they derive the Cramer-Rao lower bound of the lifetime variance with noise and demonstrate its usage with simulated and experimental data. The described approach is part of their open-domain software “Fluorescence-Lifetime TrackNTrace” and thus readily available for users.

Schneider and Schütz discuss and demonstrate the so far underused application of significance testing in single-molecule experiments. They deliver on their manuscript title, by showcasing the appropriate usage of *p*-value testing for localization cluster analysis and the proper combination of results obtained from multiple cells and/or experiments. Since cluster analysis has become its own sub-field within SMLM and the pointillistic nature of localization microscopy coupled with the iterative detection of the same molecules poses an ongoing bottleneck, their approach of attaining an overall *p*-value, testing against a random distribution of molecules has the potential to become an important standard for future SMLM cluster studies. Furthermore they present a block permutation test for single-molecule tracking data, circumventing their inherent correlation which can obscure conventional statistical significance testing.

Taken together, these articles present an overview of the broad application range of SMLM and its current trends. In light of the emerging reproducibility crisis, which includes algorithm driven high-end microscopy, it is of utmost importance to make the relevant source code or analysis software accessible to the widest possible audience. We are therefore especially pleased with the contributions to this special research topic, as virtually all presented approaches are accompanied with open-domain software and/or well documented source code (see Table 1). Thus, the work collected in this special research topic functions both as a useful, accessible resource for interested SMLM newcomers and users, as well as hopefully an inspiration for future endeavors.

**TABLE 1:** Comprehensive list of the software packages mentioned in the manuscripts in this research topic, along with their code availability destination at the time of publishing and public licensing specifications.

Authors	Software	Code	Licensing
Martens et al.	SMLMComputational	GitHub | Google Drive	GPL-3.0
Schodt and Lidke	SMLMFrameConnection	GitHub	MIT
Berberich et al.	FRCnet	GitHub	MIT
Blundell et al.	HOLLy	GitHub	GPL-3.0
Butler et al.	PALMTracer	GitHub	Custom
Chen et al.	NOBIAS	GitHub	GPL-3.0
Kutz et al.	BaClAva	GitHub	MIT
Feher et al.	KAN	Supplementary Material #2	GPL-3.0
Mancebo et al.	cross-correlation-filtering	GitHub	MIT
Thiele et al.	TrackNTrace	GitHub	GPL-3.0
Schütz and Schneider	Block-permutation method	GitHub	GPL-3.0

